# The Cryptocurrency Market in Transition before and after COVID-19: An Opportunity for Investors?

**DOI:** 10.3390/e24091317

**Published:** 2022-09-19

**Authors:** An Pham Ngoc Nguyen, Tai Tan Mai, Marija Bezbradica, Martin Crane

**Affiliations:** 1 School of Computing, Dublin City University, Collins Ave Ext, Whitehall, D09 Y074 Dublin, Ireland; 2 SFI Centre for Research Training in Artificial Intelligence, D02 FX65 Dublin, Ireland; 3 ADAPT Center for Digital Content Technology, D02 PN40 Dublin, Ireland

**Keywords:** cryptocurrencies, noise and trend effects, tick-by-tick data, network structure, community detection, COVID-19

## Abstract

We analyze the correlation between different assets in the cryptocurrency market throughout different phases, specifically bearish and bullish periods. Taking advantage of a fine-grained dataset comprising 34 historical cryptocurrency price time series collected tick-by-tick on the *HitBTC* exchange, we observe the changes in interactions among these cryptocurrencies from two aspects: time and level of granularity. Moreover, the investment decisions of investors during turbulent times caused by the COVID-19 pandemic are assessed by looking at the cryptocurrency community structure using various community detection algorithms. We found that finer-grain time series describes clearer the correlations between cryptocurrencies. Notably, a noise and trend removal scheme is applied to the original correlations thanks to the theory of random matrices and the concept of Market Component, which has never been considered in existing studies in quantitative finance. To this end, we recognized that investment decisions of cryptocurrency traders vary between bearish and bullish markets. The results of our work can help scholars, especially investors, better understand the operation of the cryptocurrency market, thereby building up an appropriate investment strategy suitable to the prevailing certain economic situation.

## 1. Introduction

The cryptocurrency market has become an attractive target for many financial investors in recent years due to its potential for rapid gains. One research topic being explored in this market is the correlation between different cryptocurrencies. Understanding how different assets interact with each other can help in portfolio optimization [1], predicting the future volatility or downturn [2] and also in observing the risk spillover that benefits portfolio diversification [3], to mention only a few.

Thanks to a network-based methodology, cryptocurrencies’ cross-relationships can be learned and observed visually [4]. The idea of this method is that it builds up a network of different objects such that the distance between two objects depends on how similar they are: the shorter the distance, the more similar the two objects are. Eventually, we can see the interaction between objects by looking at their network’s structure and analyzing characteristics of the network. Different network construction approaches have been explored in the literature, from Minimum Spanning Tree (MST) [5], k-Nearest neighbors (kNN) [6], planar maximally filtered graph (PMFG) [2] to Threshold Weighted-Minimum Dominating Set (TW-MDS) [7], to name but a few. In financial markets, normally, the similarity between two assets is measured by comparing the evolution of two corresponding price time series, one typical method to do this is Pearson correlation metric [8]. The study on correlation of traditional asset classes such as stocks, bonds, national fiat currencies and commodities has been developed a long time ago, with varying approaches invented to learn the correlation between different entities in the same market but also between different asset classes, ranging from statistical [9,10] to AI-based methods [11].

Generally, there are two common shortcomings with correlation-related studies. Firstly, one mainly uses a low-frequency dataset such as daily or monthly, and this might cause a loss of important information from each time series, hence failing to reflect their true nature [12]. This appears to be a major concern in the cryptocurrency market, since it is well-known for its high fluctuations in terms of price movement. For example, in [13], the authors show that the losses of cryptocurrencies can reach 70% within one day. Recently, in 2020, by comparing the volatility in the returns between cryptocurrency and stock markets, the authors of [14] revealed that major cryptocurrencies such as BTC and ETH have volatilities of 5.68 and 7.10, respectively, which is two-fold higher than that of S&P500 and Euro Stoxx 50 indices. Notably, Dirk et al. calculated the daily price volatility of Bitcoin from 2001 until 2021 and found that there are extremely volatile days when the volatility can hit 120% [15]. Thus, using a high frequency means that we are ignoring valuable information (e.g., the intraday fluctuations of a time series) on purpose. As a result, this can adversely affect the correlation extracted from the dataset, potentially leading to inaccurate correlation-using experiments (e.g., portfolio optimization). Secondly, researchers tend to analyze the inter-relation between different time series by using trading price values reported on a website (e.g., Coinmarket (https://coinmarketcap.com/), Yahoo Finance (https://finance.yahoo.com/)). However, this practice deliberately ignores the effects of noise and trends in financial time series, which we will describe clearly in Section 4.

Another important factor to consider is the recent *COVID-19* pandemic which forced all countries to close off borders and restrict movements for residents as well as businesses [16]. This had a strong effect on the global downturn which occurred in March 2020 as a response to governments’ efforts to control the disease spreading [17]. These historical events have been shown to disturb and devalue different financial asset classes such as stocks, bonds and also cryptocurrencies [18,19]. Instead of looking at the changes in time-series elements such as volumes, prices, returns and volatilities during the COVID-19 pandemic, in this study, we will investigate the impact of the pandemic by looking at the changes in *network structures* over time. Furthermore, based on these network’s structures, we show how we can observe the corresponding community structures via community detection methods. The results from our experiment can be used to learn behaviours of investors in different periods of time, especially during downturn times in the financial market.

From the shortcomings of existing studies and utilizing the advantage of network-based analysis, this study aims to investigate the network structure of cryptocurrencies without noise and trend effects and how this structure changes under the impact of the COVID-19 pandemic. Specifically, the research target is to answer these research questions:

- RQ1. Is there evidence of the existence of noise and trend effects in the cryptocurrency market? If yes, how do noise and trend effects influence the interactions between cryptocurrencies? What does the network structure of these cryptocurrencies look like after removing noise and trend effects?
- RQ2. Does the network structure change when the level of granularity changes? If this is the case, what level of granularity should we use to obtain the true network structure?
- RQ3. Is there evidence that historical events such as the COVID-19 pandemic and the global downturn in 2020 changed the overall cryptocurrency network structure? If this is the case, how did they change it? Moreover, is there any possibility that this change was caused by a change in investors’ investment strategy? In other words, does the way investors react to a downturn change the interactions between cryptocurrencies?

It should be noted that we are not new to the subject of time-varying cryptocurrency network structure, we merely build on work by the team of Drozdz, Watorek, Kwapien [20,21] as well as, more recently, Nie [22]. However, our work expands the existing studies since we consider the investment decisions of investors based on the observed network structure and we acknowledge the negative effect of not only trend but also noise presenting in cryptocurrencies. As suggested by Miceli [23], the trend and noise removal results in a filtered MST that better explains investment strategy and also potentially uncovers endogenous or exogenous factors that drive the price of cryptocurrencies

To solve these research questions, we use a tick-by-tick dataset which consists of 34 price time series corresponding to 34 cryptocurrencies traded on the HitBTC exchange during the period between 13 February 2019 and 6 April 2021. When it comes to network formation, we calculate the correlation between cryptocurrencies by adopting the linear similarity measurement named Pearson and then construct a Minimum Spanning Tree (MST) based on these correlation coefficients. The noise and trend removal is carried out by applying Random Matrix Theory (RMT). Community structure is found by using community detection methods. In addition, different metrics are used to analyze the network structures and support our findings.

The remainder of the article is organized as follows: Section 2 presents an overview of the relevant literature. Section 3 provides a description of the dataset. Section 4 describes terminologies, methods and preprocessing procedures. Section 5 discusses the experimental results followed by implications and hypotheses. Finally, the conclusion of this study is given in Section 7.

## 2. Related Works

### 2.1. Correlation-Based Analysis in the Financial Markets

The topic of correlation analysis has a long history in connection with stock markets throughout various historical economic crises using different correlation-calculating metrics. In [24], the authors estimated the correlation between 116 S&P500 stocks between 1982 and 2000 using Pearson coefficient. They further used MST to build up a correlation-based network in order to observe time-varying correlations based on three network measuring metrics including *normalized tree length*, *survival ratio* and *mean occupation layer*. As a result, they pointed out a large change in the network structure during Black Monday. More recently, [6] came up with a Neural Network approach to construct a graph and found a dramatic difference in the network structure during the downturns in 2008, 2011 and 2020. In [1], a Pearson correlation matrix of 200 and 400 stocks from the CSI 300 and S&P500 index, respectively, was used to find an optimized portfolio following the Markowitz optimization scheme. Instead of using Pearson method, Liu et al’s paper used an interesting alternative method Mutual Information to generate a distance metric to take account of non-linear effects in intra-day S&P stock data [25]. Other methods to estimate the correlation coefficients (i.e., Wavelet coherence, Fast Fourier Transform) and construct correlation-based networks (i.e., PMFG, threshold method) were introduced in several studies [2,11,26].

Different existing approaches to study the correlations in the stock market have been applied to digital coins. Some common conclusions from existing articles are that the cryptocurrency network changes over time but Ethereum tends to act as a central node in the whole network, i.e., it is a densely connected node [5,27,28]. A few works remedy the problem of dataset shortages that have been concerned in the traditional markets, i.e ones tended to use low-frequency data to implement their studies such as daily or weekly. However, they only account for a small portion of the existing literature. For example, Antonio et al. [29] used small frequency resolutions such as one hour and four hours and also consider daily data of 25 large market capitalized entities traded on the FTX exchange to discover the evolution of cryptocurrency network structures between different time frequencies. By using Pearson correlation-based MST, they found an increase in the complexity of networks’ shape for coarser time resolutions. In other words, cryptocurrencies converge into a bigger group as resolution increases. On the contrary, the authors in [20] using multiple timescales starting at 10 min to 360 min proposed an opposite statement that low timescales cause the network to be centralized while it is distributed and more correlated at high timescales. They used the liquidity and capitalization differences among the assets to explain this result, since cryptocurrencies with low capitalization are traded less frequently than those with large capitalization, it takes more time for a piece of market information to spread over such cryptocurrencies. Thus, they are more inclined to use longer scales. Notably, this is one of the very few studies that remove the trend effect from the original dataset. Interestingly, instead of using return time series like other researchers, a research using hourly realized volatility values was carried out to observe the risk spillover between 7 high-capitalized cryptocurrencies [3].

Different methods have been introduced to detect communities given a correlation matrix. The authors in [4] applied Louvain method on the MST of 119 cryptocurrencies to cluster potential communities. The time-varying dynamics from the community structures found suggests collective behaviour among these communities. With the communities found by the same method, the authors in [30] went one step further by using Principal Component Analysis (PCA) to find an optimal portfolio out of 200 cryptocurrencies in circulation. Another community detection method that is worth taking into consideration is Girvan–Newman, which has been adopted widely for multiple purposes such as link prediction, portfolio diversification, etc. [31,32]. A few other methods are also being used to grouping similar entities but are less popular such as Clauset algorithm, Stochastic block model (SBM), Latent Dirichlet Allocation (LDA) and Markov random field (MRF) [33]. One obstacle from existing studies is that some used a specific community detection algorithm only, raising a doubt about the robustness of the community structure. To this end, we first use the Louvain method to detect communities in our dataset and then adopt Girvan–Newman method to examine the robustness of the communities found earlier.

### 2.2. How the COVID-19 Pandemic Intervened on the Economy Worldwide

At the beginning of 2020, the economy of China started to be influenced by COVID-19, earlier than other countries. Moreover, as the world’s hub for global manufacturing and trade, immediate adverse effects on the Chinese economy resulted in global impacts [16]. Different regulations have been applied to handle the disease, such as closing national borders as well as stopping business activities across the world, strongly influencing the global economy [16]. Eventually, the global financial panic in March 2020 took place. In [18], the authors pointed out that the similarity calculated by ACC and ADCC models between the US and Chinese markets increased dramatically during the pandemic. Regarding the stock prices, when the pandemic occurred, the prices of the US and Chinese stocks decreased but started to recover again since July 2020. This trend is also true for other emerging and developed stock markets in different countries from different continents such as Japan, Germany, Australia and Canada [34]. Likewise, even less risky assets such as gold were adversely affected [35]. The increase in the correlation between different financial markets in the presence of good and bad news has been observed for some decades. In [36], the authors stated that stocks are more affected by the presence of bad news, compared to good news. Moreover, bad news has a stronger correlation in traditional markets. These results align with what happened during the COVID-19 pandemic. Although the world continued facing different COVID-19 waves afterwards, its impact on different asset classes lessened significantly [37], stock prices increased and volatilities decreased again to their original values before the pandemic [38]. Furthermore, the connectedness between different assets also experienced a major decline [39].

In [19], the authors investigated the impact of the COVID-19 pandemic on the cryptocurrency market by using daily prices of 45 well-known cryptocurrencies between September 2019 and April 2020—the majority of which are also used in our present study. In particular, they measured the stability of cryptocurrency time series using Largest Lyapunov Exponent and Approximate Entropy. All time series are divided into two parts: the first part spans September to December 2019, considered normal time, while the second spans January to April 2020, considered a pandemic period. They revealed that the pandemic increases in cryptocurrency market uncertainty as prices fluctuated significantly. Moreover, the same experiment has also been carried out on the stock market, results indicating a lower level of price fluctuations in the stock compared to digital currencies. Also on the same topic, Drozdz et al. [21] compared the Pearson correlation between the cryptocurrency market and different asset classes including stocks, fiat currencies and commodities, revealing that these conventional markets easily influence the cryptocurrency market when they are in turbulent times, while there is no significant correlation between digital currencies and other markets in normal times, given the time resolutions they used are 10 and 360 mins.

Reactions of the general public to the COVID-19 outbreak were also observed to examine its relationship with cryptocurrencies’ returns. For example, authors in [40] measured the fear of people by the frequency of occurrence of keywords *COVID-19* and *coronavirus* on Google Trends (https://www.thinkwithgoogle.com/, accessed on 4 August 2022). Thanks to the vector autoregressive (VAR) models, they compared the evolution of this fear with the stock market’s expectation of volatility VIX index (the VIX index is a measure of constant, 30-day expected volatility of the US stock market, derived from real-time, mid-quote prices of S&P500. Normally, it is calculated using the Black–Scholes formula) as well as the Bitcoin returns. They found that increases of fear can lead to Bitcoin crashes, as the correlation coefficient is −0.9. Furthermore, negative sentiment generated by coronavirus news is associated with market volatility, which is in line with other findings such as in [41]. Interestingly, some studies on the relationship between news-based sentiment and cryptocurrencies showed that, although both bad and good news cause the change in the returns and volatilities of cryptocurrencies, positive news has more effect on the volatilities and returns of cryptocurrencies in comparison with negative news [42,43,44].

Recently, network analysis in the cryptocurrency market during the COVID-19 pandemic has been carried out, with the common result being that the pandemic, as well as the global downturn, actually caused a change in the network structure of the cryptocurrency market. Specifically, cryptocurrencies tend to form bigger groups during the downtime, i.e., the number of potential clusters found in the network decreases during the downtime, with a few cryptocurrencies acting as central nodes. This topic has only been explored in a few studies to date [21,22,45,46]. Moreover, there are some gaps: (1) the lack of deep investigation of the network structure as they only consider MSTs; (2) the noise and trend effects are not removed; (3) data limitation issues.

We will address these shortcomings by doing deeper experiments on the network structure of the cryptocurrency market before, during and after the COVID-19 pandemic via a longer dataset with the effect of noise and trend removed. In addition, we will look at the way cryptocurrencies form a group during turbulent times by considering their rankings (identified by its market capitalization, the larger its maket capitalization, the higher its rank). We believe that this research can propose a better understanding of interconnections between digital currencies during standard and unstable periods. Furthermore, we also aim at understanding the investment decision of investors in different market states based on the results of community detection.

## 3. Data Description

All experiments in this study have been carried out based on a tick-by-tick price dataset (tick data are the highest resolution intraday data and consist of the sequence of each executed trade or bid/ask quote aggregated from an exchange) that was collected from the hitBTC exchange (a platform for digital asset and currency exchange to quickly and securely trade cryptocurrencies—website address: https://hitbtc.com/) from 13 February 2019 to 6 April 2021. The dataset comprises 34 cryptocurrencies with a hybrid of high and low rankings. Specifically, the highest rank is 1 (Bitcoin) while the lowest rank is 260 (FunToken), according to the price-checking website *Coinmarketcap* (https://coinmarketcap.com, accessed on 4 August 2022) in April 2021; full list in Table 1.

### 3.1. A Note on Data Sampling and Missing Data

Since price values are collected tick-by-tick, there is no fixed timescale for all cryptocurrencies leading to an inconsistency between the time series. For this reason, we re-sample the dataset by using data points at a specific timescale. In particular, we choose four different timescales, namely 30 min, 6 h, 12 h and 24 h. Each data point of a dataset is taken to be the price of the last transaction of 34 cryptocurrencies within the considered timescale. Eventually, we have four datasets corresponding to four different timescales. Table 2 shows the description of each re-sampled dataset.

Three out of four datasets have missing values with the same percentage of 0.8%. Note that a data point of a dataset is considered missing if at least one cryptocurrency does not have the price value at this data point. For each time series, instead of simply removing missing values from the time series and values from other time series from the same time, we replace missing values with the average value of the corresponding time series. This technique has been adopted in different research topics with good performance [47,48,49]. Furthermore, we notice that this does not change the statistical properties of the correlation between time series but, instead, helps to keep more information and thus the results found from conducting the experiments are more reliable and accurate.

### 3.2. Aggregational Gaussianity

Aggregational Gaussianity is considered a stylized fact in traditional financial markets. In [50], the authors observed the evolution of distributions of the IBM stock returns by looking at different levels of granularity, e.g., 30 min, one day, one week and one month, finding evidence of Aggregational Gaussianity. Another study on this topic drawing the same conclusion is described in [51]. However, these authors used different stocks and a higher set of timescales from one day to one year, showing that this stylized fact is also true for stocks at coarser time resolutions.

We investigate whether Aggregational Gaussianity exists in our log-return time series using a set of four timescales: 30 min, 6 h, 12 h and 1 day. We observe this statistical aspect by implementing three experiments: Firstly, we construct the histogram as well as kernel density estimation (KDE) for each cryptocurrency time series. Secondly, we generate the Q-Q plot, which is a popular approach to test normality for a time series [52]. Lastly, we use the Lilliefors hypothesis test for normality [53]. We obtained the following findings: firstly, although the distributions of these cryptocurrency time series have a bell curve shape at all timescales considered, they are not (from the Q-Q plot and Lilliefors test) normally distributed; secondly, however, there appears to be evidence to say that Aggregational Gaussianity exists in all cryptocurrencies used in this present study from the Q-Q plots. This result is in line with existing findings in the cryptocurrency market such as [54,55].

## 4. Research Methodology

### 4.1. Correlation Matrix Based on Pearson Coefficients and Random Matrix Theory

Given $x_{i}$ is the price time series of cryptocurrency *i*, we use its return values to find the correlation between cryptocurrencies. This is because Return values are represented as a percentage, making them scale-free and especially, stationary, which is an important requirement for many statistical tools, such as *Normalization*. Thus, we first calculate the corresponding return time series $r_{i}$ as follows [56]: $r_{i}=log\left(x_{i}^{t}/x_{i}^{t-1}\right)$, where $x_{i}^{t}$ is the price value of the cryptocurrency *i* at timestamp *t*.

Each of these return time series can be normalized as follows [57]: $\hat{r_{i}}=\left(r_{i}-\mu_{i}\right)/\sigma_{i}$, where $\mu_{i}$ and $\sigma_{i}$ are the average value and standard deviation of time series *i*, respectively.

We form a m×n matrix G such that each column represents a normalized return time series of a cryptocurrency and each row represents a timestamp. The corresponding correlation matrix C can be expressed as follows [56]: $C=\frac{1}{m}{\mathrm{GG}}^{⊺}$. In other words, each element $C_{ij}$ of C shows the correlation strength between cryptocurrencies *i* and *j* by calculating the dot product of the two normalized return time series, $C_{ij}=<\hat{r_{i}},\hat{r_{j}}>$. Such a correlation matrix is called *Pearson correlation matrix*.

It should be noted that Pearson correlation has some limitations as described in [58]. In particular, its sensitivity to outliers and inability to capture non-linear relationships both have the potential to cause misleading results. However, we believe that this correlation metric is appropriate to use in our study for the following reasons:

- Firstly, we make use of cryptocurrency returns in order to retain the statistical nature of the associated time series. While some authors have proposed addressing the nonlinearity problem (e.g., Spearman [59] and Kendall [53]), these have the disadvantage of converting rational numbers into integer rankings, with the potential to lose out on critical information from financial time series [60]. Moreover, it has been shown that rank correlation metrics also suffer from the nonlinearity issue in some cases [58].
- Secondly, Pearson has been widely applied in the existing literature, not only in the cryptocurrency market [21,22,32] but also in markets for more traditional asset classes [2,6,24]. This strongly reinforces our belief in the applicability of this method of correlation calculation for our problem.
- Thirdly, rank-based correlation metrics require independent observations. This is a known weakness of non-linear correlation methods such as Spearman and Kendall [60]. On the other hand, Pearson works well for time series with duplicate observations (because there is no requirement for independent observations), as is the case in financial time series. For example, the price of a cryptocurrency can be unchanged for a period of time.

One issue raised from this type of matrix is the question of how reliable these correlations are, in other words, whether the correlation matrix shows genuine and authentic relationships between the considered time series. Thanks to the RMT [61], this hypothesis can be examined. Particularly, given a m×n random matrix N whose elements are distributed randomly with zero mean and unit variance, the eigenvalue distribution of the correlation matrix $C_{N}=\frac{1}{m}{\mathrm{NN}}^{⊺}$ follows the Marchenko–Pastur probability density function [62] if the Quality Factor $Q=\frac{m}{n}\geq 1$ holds when the number of timestamps m→∞ and the number of features n→∞: $P\left(\lambda \right)=\frac{Q}{2\pi }\frac{\sqrt{\left(\lambda_{+}-\lambda \right)\left(\lambda -\lambda_{-}\right)}}{\lambda }$, where *P* is the Marchenko–Pastur probability density function, λ is an eigenvalue of $C_{N}$, $\lambda_{\pm }=1+\frac{1}{Q}\pm 2\sqrt{\frac{1}{Q}}$ are upper and lower limits, respectively.

From RMT, eigenvalues falling outside of $\left[\lambda_{-},\lambda_{+}\right]$ are assumed to deviate from its expected predictions [63,64]. Hence, we can use this theory to test the reliability of the relationships in our empirical data [65]. That is, if an empirical correlation matrix actually has real valuable information, it must have eigenvalues that are outside the bounds of $\left[\lambda_{-},\lambda_{+}\right]$. Otherwise, the empirical correlation matrix can be taken to contain mainly random noise. In this study, RMT has been used to test our correlation matrices. The results show that all correlation matrices are not random and contain valuable information.

### 4.2. Cleaning Trend and Noise Effects in the Cryptocurrency Market

#### 4.2.1. Noise and Trend

The cryptocurrency market is known to have a higher percentage of noise than other traditional financial markets. According to [66], the average daily signal-to-noise ratio of the cryptocurrency market is 36%, which is extremely low compared to well-established US stock exchanges such as NYSE and NASDAQ, with an average daily signal-to-noise ratio of 90%, given the considered period between March 2017 and November 2017. The noise in the cryptocurrency market might come from different sources. For instance, there is no fixed volume for a transaction to be executed at a time, so investors can freely choose the amount that they want to trade; however, this issue causes one problem, in that investors can reduce the transaction costs by splitting their budget into smaller pieces and then buy one cryptocurrency many times with different amounts of volume and price, a practice which can trigger unforseen price movements, see [67]. Furthermore, cryptocurrencies’ prices are vulnerable to “pump and dump” schemes [68], which have become pervasive recently, and also regulatory news enacted by national authorities [69]. All of these factors might intervene in the price movements of digital assets. Consequently, the correlation matrix between cryptocurrencies cannot explain their real connections as it is highly influenced by these noise factors.

On the other hand, the trend effect found in other correlated systems [70] might be found in the cryptocurrency market. Briefly speaking, a trend among cryptocurrencies means that they tend to move together in terms of price values. We notice that the majority of cryptocurrencies are created based on the protocol of leading cryptocurrencies such as Bitcoin and Ethereum (e.g., MKR, BNT, ICX, ETC and LTC) [71]. Moreover, cryptocurrencies’ prices readily fluctuate with mass media [72], causing a herding behavior [72]. Similar characteristics contribute to creating a trend in cryptocurrencies.

Generally, these phenomena might be reasons for a high-value correlation matrix of cryptocurrencies from our dataset. Thus, it is important to remove of the existing noise and trend before moving on to further analysis.

#### 4.2.2. Cleaning Method

In recent studies, different approaches have been proposed to remove the noise from a correlation matrix through modification of the corresponding eigenspectrum, e.g., Linear shrinkage [73], Eigenvector clipping [74], Non-linear shrinkage [75] and Rotationally invariant, optimal shrinkage [76]. One common obstacle for most of the existing cleaning methods is that they have parameters needing definition. This raises an obvious question: how do we choose these? It is acknowledged that a lot of effort has been made to obtain the right parameter values, i.e., the noise is removed completely without the loss of data information [77,78]. However, these optimization approaches have one issue, which is that they use the Frobenius norm in their formula, so they fail to work with outlier-containing data, a downside of the Frobenius metric [79]. On the other hand, Eigenvector Clipping distinguishes itself from others [74] as it does not require any training parameters, making its outcome robust and more reliable. Furthermore, this cleaning method is straightforward to implement, with the guaranteed efficiency as it keeps the information part, i.e., after the cleaning process, the trace of the correlation matrix remains unchanged [80]. This method has shown good performance in different studies and has been applied widely to different topics such as programming education, portfolio optimization and signal processing [70,81,82]. The outstanding performance of the Eigenvector clipping encourages us to choose this method for our cleaning scheme.

Given eigenvalues $\lambda_{1}\geq \lambda_{2}\geq \lambda_{3}\geq \ldots \geq \lambda_{n}$ and corresponding eigenvectors $v_{1},v_{2},\ldots ,v_{n}$ of our empirical correlation matrix C, we can identify k≤n such that $\lambda_{k}>\lambda_{+}$ and $\lambda_{k+1}\leq \lambda_{+}$. The Eigenvector clipping defines the denoised correlation matrix $C_{denoised}$ by [83]:

(1) $$C_{denoised}=\Sigma_{i=1}^{n}\lambda_{i}^{*}v_{i}v_{i}^{⊺},\lambda_{i}^{*}=\left\{\begin{array}{c} \frac{\lambda_{k+1}+\lambda_{k+2}+\ldots +\lambda_{n}}{n-k},\forall i\geq k+1\\ \lambda_{i},\forall i\leq k \end{array}\right.$$

Equation (1) uses the same eigenvectors as C but modifies their corresponding eigenvalues such that those greater than $\lambda_{+}$ remain unchanged while the rest will be replaced by their average value. Notably, although small eigenvalues are replaced, the trace of the denoised correlation matrix is equal to its origin.

Regarding the trend effect, it is explained by the first eigenvalue and eigenvector, referred to as “market component” [83]. The market component is proved to influence the outcome of the correlation matrix. In particular, it is involved in all interactions observed from the correlation matrix due to its enormous amount of information, consequently, lessening the performance of clustering algorithms [84]. Thus, removing this component is a necessary step to clean the trend effect so that a greater portion of the correlation can be explained by components that affect specific subsets of the cryptocurrencies and, hence, facilitate clustering algorithms to find dissimilarities across clusters. A cleaned correlation matrix $C_{cleaned}$ is obtained by subtracting the market component from the denoised correlation matrix:

(2) $$C_{cleaned}=C_{denoised}-\lambda_{1}v_{1}v_{1}^{⊺}$$

We found that the connections between cryptocurrencies decrease greatly without noise and trend effects: large cryptocurrencies such as Bitcoin, Ethereum and Ripple do not see to affect the cryptocurrency market as they did before the cleaning process, since there is no strong connection between them and other cryptocurrencies. This result is in line with [70], where the Eigenvalue Clipping method was also used to clean the education-related correlation matrix.

### 4.3. Distance Matrix and Its Minimum Spanning Tree

Although the correlation coefficient can explain some aspects of the relationships between cryptocurrencies, it is not a metric [85]. Thus, the connections learned from the correlation matrix lack topological characteristics because they are not placed in a metric space [85]. To tackle this issue, a concept named *Distance Matrix* has been introduced to replace the correlation matrix.

Let **D** be a distance matrix deriving from $C_{cleaned}$, then:

(3) $$d_{ij}=\sqrt{2*\left(1-c_{ij}\right)}$$

where $d_{ij}\in \left[0,2\right]$ is an element of **D**, with 0 indicates the complete similarity between 2 nodes while 2 indicates the complete difference between 2 nodes. From the Equation (3), we can prove that: (1) $d_{ij}\geq 0$, (2) $d_{ij}=0$ if i=j and (3) $d_{ij}=d_{ji}$, i.e., the requirements of a metric are satisfied [85]. By using the distance matrix, we can derive a network (graph) of cryptocurrencies (nodes) with a specific topology, where similar cryptocurrencies are close to each other and cryptocurrencies with different behaviors are far away from each other, the link (edge) between each pair of cryptocurrencies is their distance value. Thanks to this topology, different communities of cryptocurrencies can be observed.

One problem with this type of network is that it is dense. That is, for a set of *N* nodes, the corresponding graph deriving from D has $\frac{N\times (N-1)}{2}$ edges such that each vertex connects to all other vertices. To reduce the complexity of the network, we use a Minimum Spanning Tree (MST) [86], which refers to a special tree from the graph that links all vertices together in which its length is minimal. Particularly, it reduces the amount of redundant information since it only keeps the N−1 most important edges, i.e., N−1 shortest edges that are well connected. MST stems from graph theory and is applied widely to different fields [4,87,88], especially in financial markets [89,90,91]. To exploit the useability of MST, the dynamics of community structures in the stock market are observed by Huang et al. [92] with the dataset split into consecutive smaller periods and a MST constructed at each of them. Thus, the characteristics of a financial network can be captured by observing the evolution of MSTs. More recently, the cryptocurrency market was introduced and attracted a number of investors, and the demand for exploring the correlation between cryptocurrencies thereby emerged. However, this topic is rather new and needs more studies to be implemented [4,93].

There are two famous algorithms to find the MST, namely Prim [93] and Kruskal [94]. While both methods show good performance, Kruskal seems to be better in terms of time complexity. A comparison between the two from [95] shows that the prior works well with a big network, while the latter is dominant when the network is small, which is appropriate for this study as we have only 34 cryptocurrencies. Moreover, Kruskal is used more often in finance-related topics compared to other approaches [96,97,98], which strengthens the reliability of the algorithm. With these advantages, we choose Kruskal for this study.

### 4.4. Community Detection in the Cryptocurrency Market

Given a MST from the distance matrix D, different communities are formed and can be recognized clearly, i.e., cryptocurrencies belonging to one community have short distance edges among them and the distance between two others in two different communities is longer than any edges of these two communities. However, there are less common cases in which some nodes are scattered between communities, or it is not visible from the graph how close the two communities are. This issue motivates us to further analyze the MST to optimize the clustering result using several community detection methods which have been developed [99,100,101,102,103]. Of these, the Louvain method is applicable across a wide range of domains [104,105,106,107]. Thus, we apply this method to our MST in order to obtain optimal communities. Theoretically, Louvain is an optimization problem that uses *Modularity* to measure the density of links inside communities compared to links between communities. The target of Louvain is to minimize the Modularity measure, which means that different authentic communities are clustered very tightly [108].

However, it is not convincing just to show results from one method only, as the community structure of a network might be just random. To overcome this issue, we also adopt another commonly used method named Girvan–Newman, which removes edges from the original graph one-by-one such that the edge having the highest number of shortest paths between nodes passing through it is removed first. Eventually, the graph breaks down into smaller pieces, so-called communities [109].

If the results proposed by these two community detection methods are similar, it implies that the relationship of the cryptocurrencies as well as their corresponding community structure are reliable and reflects their genuine characteristics. The results after applying these methods are shown in Section 5.

### 4.5. Time Window Division

Given the dataset described earlier, one important question about constructing a network structure in these cryptocurrencies is how to split the dataset into different consecutive periods. This is because a network structure corresponding to each period of time should be able to explain what has happened to the cryptocurrencies throughout that time, i.e., there must be a reason behind this topological structure. If we divided the dataset randomly, we could not capture important historical events at a specific period. As a result, the topology we found would be meaningless in the corresponding time window. To this end, we must select time windows rationally. We note that our dataset contains the period of the COVID-19 pandemic as well as the global downturn 2020. From the literature in Section 2, we see these historical events actually adversely influenced the financial markets. Thus, we postulate that the COVID-19 pandemic is a reasonable milestone to separate our dataset.

To verify the pandemic’s impact on the global economy and thereby choose the right time windows for the dataset, we consider the movements of four different factors. Firstly, the attention to the COVID-19 pandemic, as measured by the frequency of COVID-19-related keywords searched on Google Trends. For this factor, we use two keywords including COVID−19 and coronavirusdisease19. Secondly, we use the VIX index to observe fluctuations of the stock market, this index starts at 0 for no upper bound and a higher value implies that the stock market has stronger fluctuation. Thirdly, we also observe the prices of the S&P500 index, representing the US economy. Lastly, the growth rate of the world’s GDP is used as a proxy for the development of the global economy in general.

**Figure 1:**
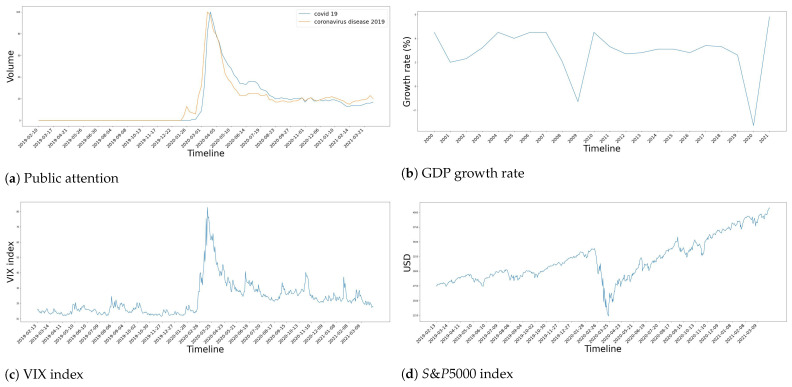
The reaction of general public and global economy to the COVID-19 pandemic. Four factors are considered: (**a**) Worldwide attention to the pandemic, (**b**) Global GDP growth, (**c**) VIX index, (**d**) S&P500 index.

Figure 1 visualizes these aforementioned factors. From Figure 1a, people started to worry about this disease in January 2020. However, it was not until March 2020 that the COVID-19 pandemic actually caught the attention of people worldwide, as the volume of searches for COVID-19-related terms quickly peaked. This remained a topic of interest until July 2020. Furthermore, March 2020 was the month in which a pandemic-induced economic recession first occurred, seriously affecting the economy of nations worldwide. This effect is shown in Figure 1b–d. In particular, the GDP’s growth rate decreased by 3.3% in 2020, which is the highest decrease ever, even worse than the Great Recession in 2007–2009 [110]. Simultaneously, the stock market fluctuated dramatically, which can be seen via the VIX index and the S&P500 index, both of which experienced a significant fall during March 2020. However, the economy started to recover afterward, the stock market became less fluctuated and the S&P500 index regained its original pre-pandemic value in July 2020.

Consequently, we split the 784 days from 13 February 2019 to 6 April 2021 into three time windows which correspond to three different stages, including normal time, downturn time and recovery time. The details for these time windows are shown in Table 3.

## 5. Experimental Results and Discussion

This section sets out our three research questions. We will first examine the impact of noise and trend effects on the correlation between cryptocurrencies as well as their corresponding topological structure. Then, we observe the evolution of the structure according to the levels of granularity. Finally, the results from these two experiments will be used to construct the right network structure. Consequently, the corresponding community structure is identified, which is used to learn the investment decisions of crypto investors during the COVID-19 pandemic.

We note that all calculations in our study are implemented using *Python* programming language (version 3.7.14, designed by Guido van Rossum, Centrum Wiskunde & Informatica (CWI), The Netherlands). Regarding network-related calculations (e.g., network construction and network-involved metrics), we utilize the *networkx* (https://networkx.org/) package incorporated into Python.

### 5.1. The Response of Network Structures to Noise and Trend Effects

Given the fact that there are noise and trends in the cryptocurrency market, we examine whether these factors affect the cryptocurrency network structure. Since we have four datasets corresponding to four timescales (e.g., 30 min, 6 h, 12 h and 24 h), we use both metric-related methods and visualization for all available datasets to discover the discrepancy between original and cleaned (after removing noise and trends) datasets.

To show the difference between two network structures, we choose two such metrics to measure the connection strength in a network of cryptocurrencies:

- Residuality Coefficient [93]: This compares the relative strength of the connections above and below a threshold distance value. In this experiment, we use the highest distance value ensuring connectivity of the MST as the threshold, denoted *L*:
  (4) $$R=\frac{\Sigma_{\left[d_{ij}>L\right]}d_{ij}^{-1}}{\Sigma_{\left[d_{ij}\leq L\right]}d_{ij}^{-1}}$$
- MST-based mean distance [111]: this calculates the average distance of the MST:
  (5) $$M=\frac{1}{N-1}\Sigma_{d_{ij}\in MST}d_{ij}$$

An increase in these means that cryptocurrencies are further from each other. By contrast, cryptocurrencies are closer to each other if these metrics decrease. Note that although both metrics are used to examine the connection strength of cryptocurrencies, the Residuality coefficient is known to be more vulnerable to the links between cryptocurrencies in different groups, i.e., if the connection strength between cryptocurrencies in different groups increases, the Residuality coefficient will decrease dramatically, and vice versa; the connections between cryptocurrencies within one group do not affect the Residuality coefficient much [112]. On the other hand, Mean distance is more vulnerable to the links between cryptocurrencies belonging to one group, as it mainly uses the connections within a group to find the average value and ignores the connections between different groups [111].

**Figure 2:**
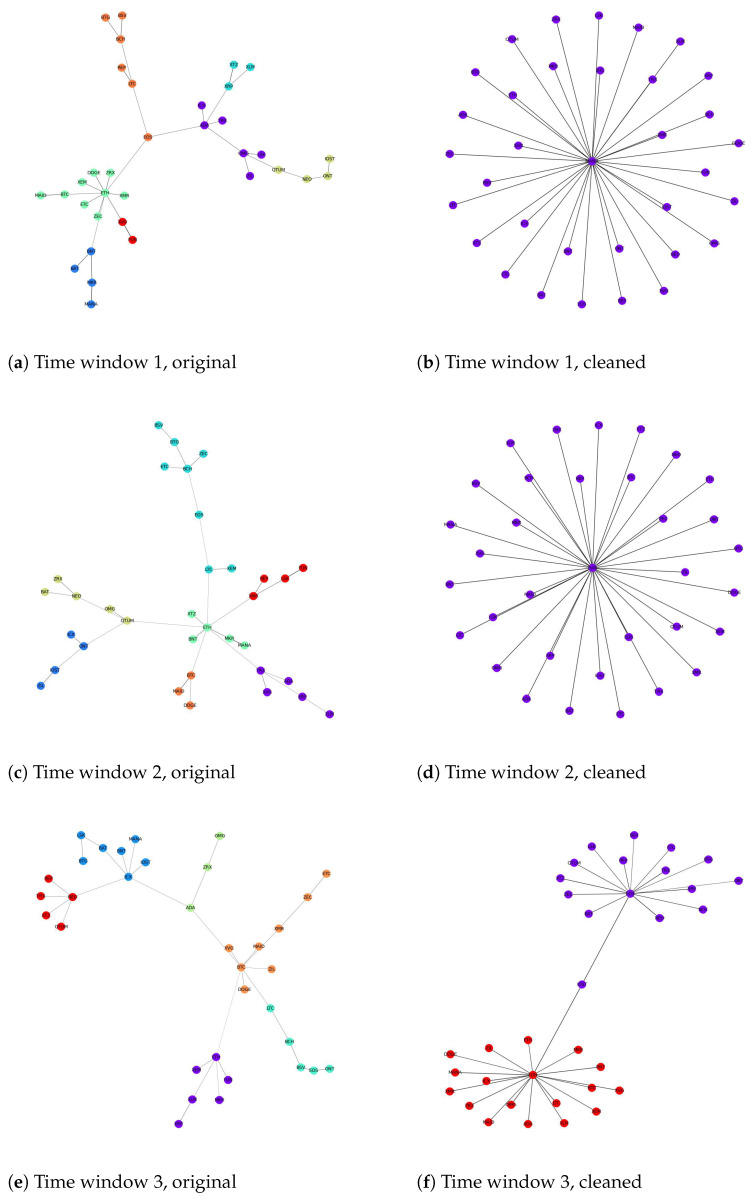
Cryptocurrency network structures using daily data. For each time window, Louvain method is applied to both original and cleaned data to detect existing communities. The illustrations on the left and right hand side are for the original and cleaned data, respectively, for 3 time windows referring to normal, downturn and recovery times, respectively.

Table 4 shows the results of the two metrics using different levels of granularity. It is clear that both Residuality coefficients and Mean distance values increase significantly when the effects of noise and trend are dismissed. This phenomenon remains unchanged in different timescales, implying that this is a genuine characteristic of the cryptocurrency market. Furthermore, a visualization of network structures before and after cleaning is shown in Figure 2 to reinforce our finding. As can be seen, the topological structure changes after the noise and trends are removed. Moreover, what happens in each time window is that the number of communities decreases after removing these effects. From these figures and illustrations, we can conclude that the connections between cryptocurrencies are caused mainly by the noise and trend effects. That is, these factors result in different cryptocurrencies becoming closer to each other and forming a group. This phenomenon can be explained by low values for Residuality coefficients and Mean distance values in the original data compared to the cleaned data. A value less than unity of the prior metric means that there are few connections greater than the threshold *L*. Moreover, a small value of the latter metric means that cryptocurrencies within a group are closer to each other. In summary, each group of the network is compact with strong links inside, which helps the community detection algorithm to easily cluster them. In other words, the difference between different groups is clear because the links between different groups are weak, i.e., the ones greater than *L*. However, after cleaning the correlation matrix, cryptocurrencies that are closely related to each other through noise and trend become further away, i.e., the strong links between some cryptocurrencies are broken. This causes our metrics to increase dramatically, which means that the network structure starts to expand, forming a sparse network. For example, the Residuality coefficient of the second time window in the 30 min original data is 0.28, while it is 20 times higher after cleaning the effects of noise and trends. This fact is also true for the rest of our dataset. The result is in line with [20]; these authors did not consider the noise effect but, with the removal of trends, they found that the correlation between the 80 most liquid cryptocurrencies from 1 January 2020 to 1 October 2021 decreased.

### 5.2. Real Network Structures in Different Levels of Granularity: An Experiment on Cleaned Data

In this section, we will construct the network structure of 34 cryptocurrencies removing the effects of noise and trends. By doing this, we can look at the evolution of network structures at each timescale over time and, of greater interest, the differences in the network structures between different timescales. Note that community detection results found by using Louvain algorithm are also included in these networks. The results of this experiment can shed light on the influence of timescales on cryptocurrencies’ connections and what timescale should be used for cryptocurrency-related analysis.

#### 5.2.1. The Evolution of the Cryptocurrency Network According to Timescales

**Figure 3:**
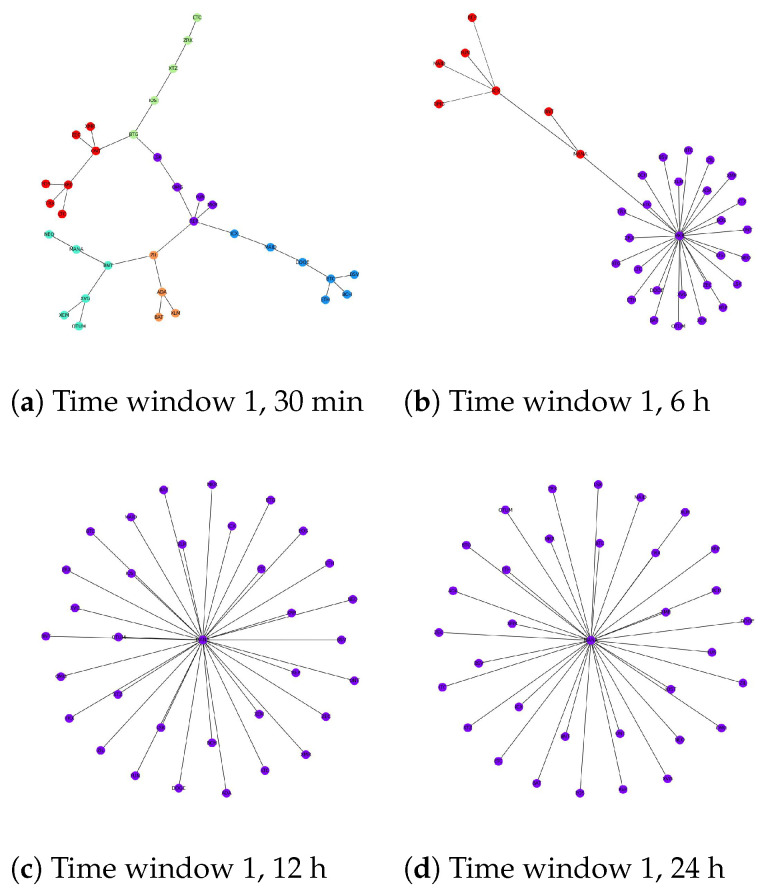
Network structure for the first time window, community detection is applied using Louvain method. Four different timescales are used, e.g., (**a**) 30 min, (**b**) 6 h, (**c**) 12 h, (**d**) 24 h.

**Figure 4:**
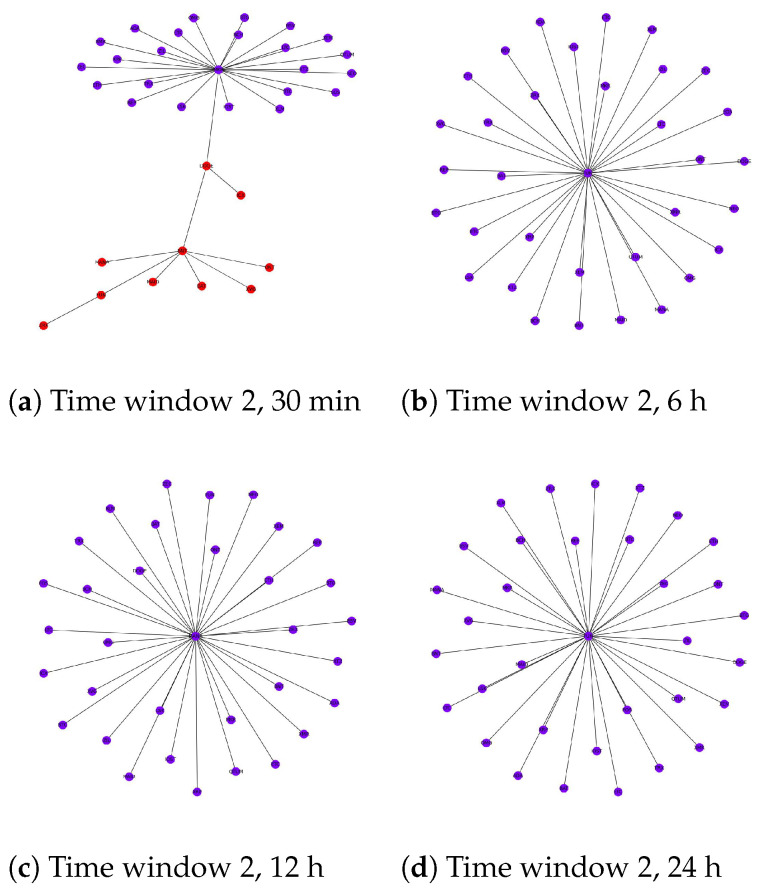
Network structure for the second time window, community detection is applied using Louvain method. Four different timescales are used, e.g., (**a**) 30 min, (**b**) 6 h, (**c**) 12 h, (**d**) 24 h.

**Figure 5:**
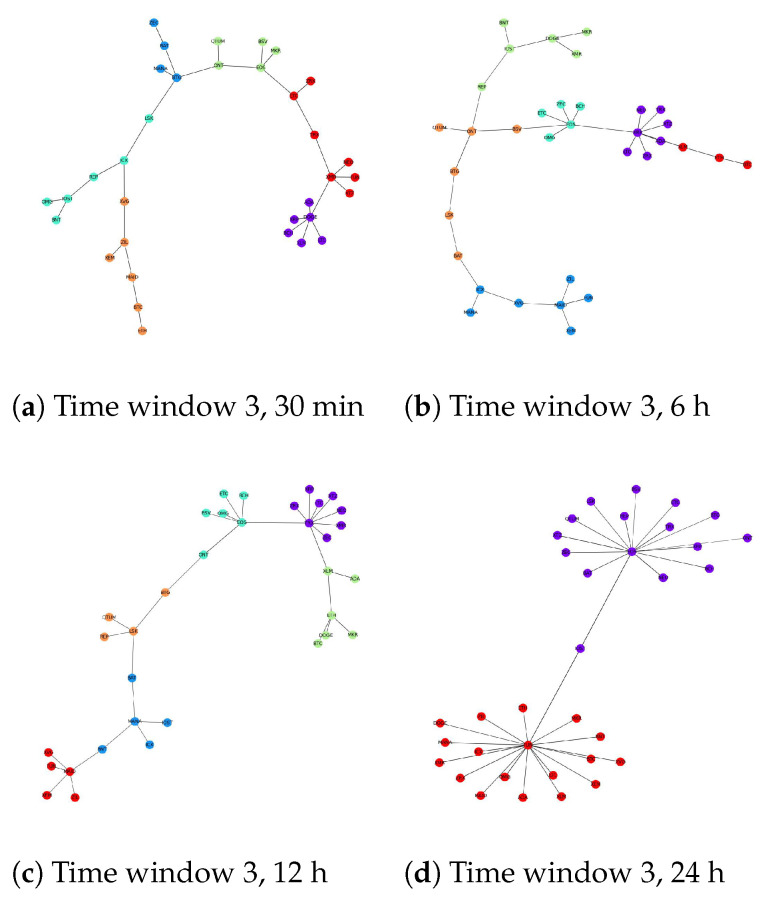
Network structure for the third time window, community detection is applied using Louvain method. Four different timescales are used, e.g., (**a**) 30 min, (**b**) 6 h, (**c**) 12 h, (**d**) 24 h.

Figure 3, Figure 4 and Figure 5 show the results of network structures along with detected communities using the Louvain method with each figure representing a different time window. For each window, four network structures corresponding to four different levels of granularity are displayed. One obvious statement that can be made from the illustrations is that the community structures at each level of granularity change over time. Additionally, if we consider different levels of granularity at the same time, the number of detected communities tends to decrease when the timescale becomes more coarse-grained. For large timescales, such as 24 h, cryptocurrencies build up big groups with few cryptocurrencies acting as central nodes that link directly to the remainder. For example, in Figure 3d, MANA acts as a central node that links all other cryptocurrencies together. This explains why community detection techniques cannot distinguish several subsets as the network in this case is naturally one group. Figure 4d shows a similar pattern, while in Figure 5d there are two central nodes that create two big groups with relatively similar sizes. To this end, with low-frequency data, we expect we can predict the long-term trend of cryptocurrencies in the future by looking at the central nodes from their corresponding community structures. If this is the case, it will be very beneficial for investors who choose a long-term investment. However, this behaviour requires deeper investigation and will be the subject of further research.

We notice that the difficulty of detecting communities in this market increases with the timescale length. In other words, cryptocurrencies are more likely to belong to the same community if we just look at their price values at a high level of granularity such as daily. Thankfully, it can be explained based on the nature of the cryptocurrency market. In particular, the cryptocurrency market is well-known for its high volatility compared to other traditional asset classes such as stocks, bonds and commodities [113,114,115,116]. In [117], the authors used 5 min data of Bitcoin prices traded on three different exchanges, Kraken, Bitstamp and Btcbox, during the period between 2017 and 2021 to calculate the realized volatility (the assessment of variation in returns for an asset by analyzing its historical returns within a defined time period) of this most stable and popular cryptocurrency. The results showed that although Bitcoin is the most valuable and trustworthy cryptocurrency, its volatility fluctuates from 4.8 to 7.5. By contrast, with the same level of granularity, the stock market seems to be more stable, as the realized volatility stood at roughly 0.58 during normal times [118] and increased to just around 1.0 during the COVID-19 pandemic [119]. These facts suggest that the cryptocurrency price fluctuations are dramatic even within a 5 min period. Consequently, using a low-frequency dataset such as 12 h or 24 h appears to cause a loss of important information that influences the results of analysis. This problem has also been described in earlier studies such as [12]. However, existing studies mainly focused on daily data to detect communities in the cryptocurrency market.

In this study, the loss of information by using large timescales including 6 h, 12 h and 24 h makes judging the correlation between different cryptocurrencies unclear. As a result, it affects the corresponding MST which can be seen in Figure 3, Figure 4 and Figure 5. Ideally, we would like to use a dataset that is as fine-grained as possible. Unfortunately, our experiments show that for frequencies lower than 30 min, there are a huge amount of missing values as some cryptocurrencies are not traded frequently [120], thus requiring their removal or imputing a value. This adversely affects the correlation between time series and impacts our analysis. Finally, we choose a 30 min dataset for further experiments.

#### 5.2.2. Louvain vs. Girvan–Newman for Community Structure Detection

The Louvain method is our main technique for detecting communities but we also use the Girvan–Newman method to double-check the communities found. The *v*-measure gives the similarity between these two methods [121], shown in Table 5. This metric ranges from 0 to 1 such that 0 indicates a complete dissimilarity between two graphs while 1 indicates a complete similarity. We found that the *v-measure* in all cases is high with the lowest value of 0.82 from the third time window in the 6 h dataset in Table 5. That is, the Louvain method proposes similar results as Girvan–Newman. Thus, the communities found by Louvain are reliable for use in further analysis.

### 5.3. Analysis of Investors’ Investment Decisions Based on the Time-Varying Network Structure

#### 5.3.1. The Changes in Crypto Network Structure during Times of Crisis

To observe the growth of the network structure over time, we use *Degree Assortativity Coefficient* [122] and *Average Betweenness Centrality* [3]. However, these metrics fail to tell us the similarity between two networks. Thus, to statistically compare the topological change between two networks, we use three more metrics, including *v-measure*, *Degree centrality* [26] and *Eigenvalue method* [123,124].

Table 6 shows results of *Betweenness Centrality* and *Degree Assortativity*. Immediately, we can see that there is a huge change occurring in time window 2, which corresponds to the turbulent time caused by the pandemic on both metrics.

Regarding the *Betweenness Centrality*, this metric decreases from 0.15 in time window 1 to 0.05 in the next period before going back to its original value prior to the pandemic outbreak (time window 1). A reasonable explanation for this movement is that the network structure of the cryptocurency market during normal times appears to have a dispersive tendency with the whole network divided into multiple small-size groups such that each group share common characteristics. However, during COVID-19, these synchronize to form a big group. Thus, the number of groups decreases while the size of each group increases. This might be a consequence of an increase in the connectedness of cryptocurrencies during the pandemic, as shown in many research papers [11,27,45]. In the recovery time, however, the network started to divide into smaller parts again, perhaps because the cryptocurrency market overcame the most connected period and started to go back to its normal behavior.

The *Degree Assortativity* results strongly support those of the *Betweenness Centrality*. In particular, a negative value shows that high-degree nodes are more likely to link to low-degree nodes, which means that each group in the network has one node acting as a central node connecting to the rest. While the values in time window 1 and 3 are approximately the same, time window 2 shows a decline by nearly 50 percent. This indicates that the number of connections between high-degree nodes and low-degree nodes increases, i.e., the network forms big groups with a large number of leaf nodes in each group.

We notice that this time-varying structure is similar to what have been shown in works of Drozdz et al. [20,21], who stated that the market has a distributed-network topology or a hierarchical-network topology in which no node dominates the network during normal times. However, it becomes more centralized during the pandemic and started to distribute as this turbulent time is gone. More recently, another work proposed by Nie also confirmed the same result [22].

Table 7 shows results of the three similarity metrics for different time periods: *normal time* (time window 1), *downtime* (time window 2) and *recovery time* (time window 3). Each values shows the similarity between two time windows. For v−measure, the higher the value is, the more alike two networks are. On the other hand, for the remaining values, a lower value indicates that two networks are more similar.

The differences between time window 2 and the other two time windows are very clear. In particular, the v−measure between time window 1 and 3 is 0.32, meaning that communities found in time window 3 hold roughly one third of characteristics from time window 1’s communities. By contrast, v−measure values between time window 1 and 2 as well as between time window 2 and 3 are negligible, standing at 0.04 and 0.02, respectively. Additionally, for the topological structures of MSTs, the other two metrics also show the same principle since time window 1 and 3 share common characteristics and the similarity degree of other cases are nearly zero. Remarkably, the Eigenvaluemethod shows a significant divergence of time window 2 with others, as shown in Table 7.

The severe pandemic and the global downturn of March 2020 together seem to have actually changed the way cryptocurrencies interact with each other. The changes of these interactions have created new communities and broken down old ones, i.e., some cryptocurrencies become closer to each other while others moved further away from each other due to the COVID-19 pandemic and the economic recession. Eventually, the topological structure during this turbulent time shows completely different patterns compared to the periods when the global market is stable. Furthermore, we noticed that the community structure started to recover back to its pre-COVID-19 levels after June 2020, which coincides with the time the global economy recovered and the COVID-19 pandemic had less impact. During this time, some characteristics of the network structure reappeared that are similar to the structure during the normal time (it is obvious that these structures are not fully similar because they change over time, as proven in previous sections and, in addition, after June 2020, the global economy started to recover, but not as well as in the past, and the pandemic still had an impact on the economy worldwide to some extent). This is why the v−measure between time window 1 and 3 increased significantly and the corresponding differences measured by Degreecentrality and Eigenvaluemethod are very small. The community structures for the three time windows are shown in Figure 3a, Figure 4a and Figure 5a.

#### 5.3.2. Learning the Investment Decision of Crypto Traders Based on Ranking Distribution

The ranking of a cryptocurrency is measured by its market capitalization (a multiplication between the number of coins in circulation and the current market price of a single coin). We obtain cryptocurrencies’ ranking on the https://coinmarketcap.com website (accessed on 15 August 2022).

We use this characteristic of cryptocurrencies to examine how they are distributed in each community of the cryptocurrency network. More importantly, we will have a look at the way cryptocurrencies form groups during different phases of the global economy by observing the distribution of ranking in each group between different periods of time.

Table 8 summarizes the results of community detection using the Louvain method. For each period of time, the found communities are listed with a set of cryptocurrencies and corresponding rankings belonging to each of them. We found that during the normal time, there is a mix between high-ranking and low-ranking cryptocurrencies in each community. For example, group 6 has a size of 7 including top-ranking cryptocurrencies such as BTC, ETH and BCH, while also having very low-ranking ones such as MAID and ICX. We pay more attention to communities found in the downturn time. At this phase, we recognized that the community formation of these cryptocurrencies seems to be dramatically different from the previous period. In particular, there are only two communities found during this period, while the other has six. More importantly, there seems to be a separation between high-ranking and low-ranking cryptocurrencies, because the majority of top-ranking cryptocurrencies belong to one group while the majority of low-ranking cryptocurrencies are in the other. Additionally, by comparing these results with the period of recovery, we noticed that this period shares common characteristics with both normal time and downturn time. Specifically, after the downturn time, cryptocurrencies started to separate from each other; this can be seen by looking at the number of communities during this time. There was an increase from 2 to 6, which is equal to the normal time case. While the majority of communities show a mix between high- and low-ranking cryptocurrencies, there are two communities that are similar to the downturn time: group 4 with all high-ranking cryptocurrencies and group 5 with all low-ranking cryptocurrencies.

**Figure 6:**
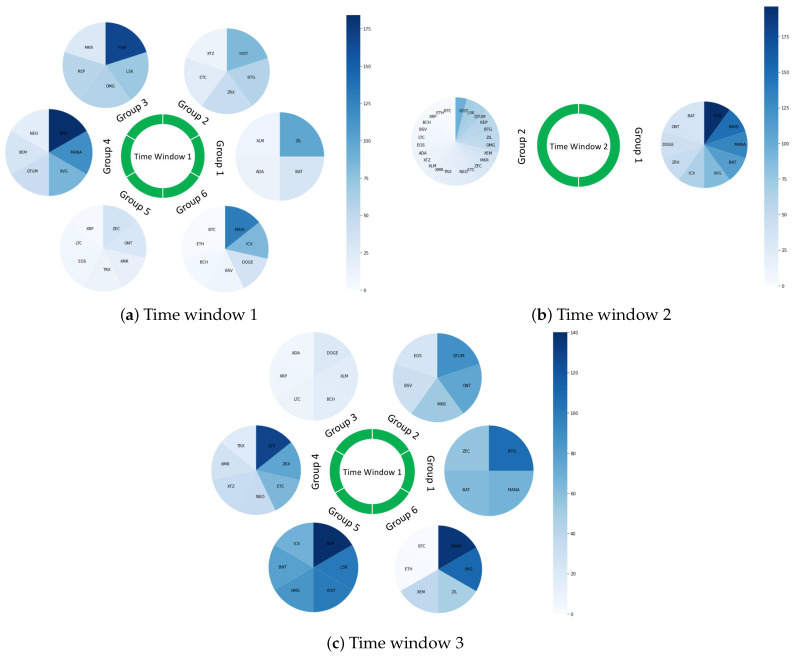
Cryptocurrency’s rankings distributions in three different phases of time. Each community is represented by a circular shape while the rankings of cryptocurrencies in this community are given by the blue color intensity, i.e., the darker the blue, the lower the cryptocurrency’s rank.

Figure 6 shows the distribution of cryptocurrencies’ rankings in three different phases of time. We use this visualization to show readers the changes of ranking distributions in a clearer and easier manner. Each community is represented by a circular shape while the rankings of cryptocurrencies are represented by the intensity of the blue color, i.e., the darker the blue, the lower the cryptocurrency’s rank. Figure 6b shows that the circular shape of group 1 is clearly darker than that of group 2. On the other hand, there is a combination of both bright and dark blue in the majority of cases in two remaining sub-figures. Notably, Groups 3 and 5 in Figure 6c show a clear difference from the rest.

When it comes to these results, investors’ investment decisions can be considered as potential explanations for the time-varying community structure. During normal times, i.e., when the financial market is stable and there is no major event occurring that impacts wider society, investors show a non-herding behaviour. That is, their decision for investing in a cryptocurrency is based on their own market analysis and is not influenced by other investors’ choice. This might push up the vibrancy of the cryptocurrency market where a large number of coins with both high and low rankings are traded. As a result, there is a diversification in terms of rankings in each community. Empirically, it is found that there was no herding behavior before the pandemic. In particular, Larisa et al. in [125] used hourly price time series of multiple exchanges such as Binance, Bitbay, BitFinex, Coinbase and major cryptocurrencies including BTC, LTC and ETH to find the existence of herding before the start of COVID-19. Based on the *Cross Sectional Absolute Deviation* model, they found that the herding behavior was free during this time. By contrast, during a turbulent time, investors are panicked by the fluctuations of cryptocurrencies’ price as well as being bombarded by bad news that strongly affect their investment. Different studies have been carried out to investigate the investors’ behavior since the onset of the COVID-19 outbreak. Generally speaking, these reached the same conclusions: that the pandemic actually increased herding behavior in the cryptocurrency market. In [126], the authors used 43 cryptocurrencies with large market capitalization between 2013 and 2020; they found that investors in the cryptocurrency market follow the consensus and the impact of coronavirus media coverage is significant on the herding behavior. In particular, news related to the coronavirus increases fear and affects the behavior of investors reducing appetite for risk. Consequently, investors disregard their private information and follow others’ investment decisions. However, the impact of media is reduced when the market returns to a normal phase. This is in line with different studies that use different datasets and time periods [125,127,128]. More importantly, the way investors show herding behavior is that they tended to invest in the major and most-tradable cryptocurrencies [27]. This can be explained by the fact that high-ranking cryptocurrencies are more mature so they are more stable than the rest and are more likely to retain value under the uncertainty of the global financial market, causing a bias from investors [129]. Consequently, major cryptocurrencies were seen to increase in terms of trading volume and act as a store of value during the turbulence times [130]. In other words, there was a risk aversion occurring after the pandemic outburst as described in [72]. Eventually, all high-ranking cryptocurrencies belonged to one group.

When it comes to low-ranking cryptocurrencies, we notice that cryptocurrencies with the lowest rankings in our dataset belong to another group. This might be because they receive the same treatment from investors during the downturn time, so they have the same trend. One possible reason for this is that low-ranking cryptocurrencies are less likely to be used as an investment option during the downturn time because they have negligible value and bring more risk to investors. Instead, they are mainly used for other purposes, such as paying transaction fees, as currency for a smart contract or simply a token on a cryptocurrency platform used to access applications [27]. This seems reasonable as the pandemic stopped in-person interactions. Hence, they had to complete all work remotely. In this case, cryptocurrencies and blockchain technology are extremely useful since they thrive under the proposed online environment to resume working activities worldwide and also bring benefits to users. Being used for the same purpose causes a similarity between these cryptocurrencies.

All findings that we have shown earlier help us to explain the community structure in time window 3, which corresponds to the recovery period. During this time, the concerns about the pandemic started to decrease, meaning that not only cryptocurrency but also other traditional assets recovered with investors’ newfound positive attitude bringing them back to normal trading. Crypto traders started to diversify their portfolio by investing in different low and high market-capitalized assets and making their own decisions [126]. However, one remarkable phenomenon that is worth taking into consideration is ] risk-taking behavior. A piece of research implemented by Christoph et al. [131] used 100 return time series of risky stocks to conduct a survey related to the investment behavior of professional market traders. The responses of more than 800 participants revealed that a number of investors underestimate risk after prolonged exposure to high risk, as they become accustomed to the uncertainty of the economy. Thus, they go back to investing in risky assets or even engage in more risk-taking to gain more profits. This tendency explains the similarity in the community structures between time windows 1 and 3. However, as we can see, there exists one group with high-ranking cryptocurrencies and one group with low-ranking ones as a result of the risk aversion of a portion of investors after the great shock caused by the pandemic.

## 6. Limitations and Future Works

### 6.1. Limitations

Although the tick-by-tick dataset used in this study is large, which strengthens the results of the experiments, the number of cryptocurrencies should ideally be higher so that we can draw firmer conclusions regarding the cryptocurrency market (e.g., whether the results generalise for large-cap and small-cap crypto assets). This will be the subject of future work. Secondly, while 30 min granularity has been found to suffice for our calculations, it would be better if we could use a lower level, say 15 min or even finer. Unfortunately, some cryptocurrencies are not traded regularly causing a lot of missing values at these timescales. This will also be the subject of future work.

There is also a concern with respect to the use of Pearson correlation for clustering problems. In particular, although this correlation metric has been applied widely in the existing literature and proposed various findings in the financial markets [2,21,22], it is sensitive to outliers [58] and cannot capture non-linear relationships that might cause misleading results [25]. Consequently, this adversely affects the clustering results. Indeed, these issues are also observed in other correlation metrics such as Spearman and Kendall [58]. Furthermore, we noticed that the results of clustering vary significantly by using different correlation measuring methods. Thus, it is necessary to deeply investigate different methods for a specific research task and analyze the results from each of these methods. Indeed, the creation of new approaches for calculating correlation coefficients that overcome the current limitations needs to receive more attention.

### 6.2. Future Works

Understanding how cryptocurrencies are correlated with each other sheds light on portfolio optimization. Based on the outcome of this study, we can take one step further by constructing and comparing the portfolio optimizations at different phases of the market, i.e., during bear and bull market periods. Therefore, the unique characteristics of an optimized portfolio at different market phases can, in theory, be learned and analysed. Secondly, we have noted that different network structures can be observed for a number of exchanges. Thereby, a comparison between them can be made. Another future plan which is worth taking into consideration is to observe the correlation using different techniques. For instance, we are aiming to use mutual information, which is successfully applied in [25], to estimate the correlation between two cryptocurrencies. This method can overcome obstacles from popular linear and non-linear methods since it can measure the non-linear correlation while allowing the existence of non-monotonic relationships. Lastly, we have noticed that the network structure of low-frequency data behaves differently to that of high-frequency data. We remark that we can expect to learn the long-term characteristics of cryptocurrencies based on this structure which could be potentially beneficial for investors who choose to make a long-term investment decision.

## 7. Conclusions

This research aims at answering three questions related to cross-correlations in the cryptocurrency market: Firstly, how do noise and trends in cryptocurrencies influence their cross-correlations and then the corresponding network structure? Secondly, what level of granularity should we use? Lastly, is the dramatic change in the cryptocurrency network structure during the pandemic caused by investors’ investment strategy? We firstly analyze the effect of noise and trend in cryptocurrencies on their cross-correlations and then remove these factors thanks to Random Matrix Theory and Market Component. Four sub-datasets with different levels of granularity including 30 min, 6 h, 12 h and 1 day are created from the original tick-by-tick data to examine the importance of choosing the right frequency resolution. Then, we use MST to construct a correlation-based network and detect different potential communities by using Louvain and Girvan–Newman algorithms. We found that the correlations between cryptocurrencies are mainly caused by noise and trend effects, which might lead to a big problem for the traders’ investment strategy because investors might be fooled by looking at the counterfeit relationship. It is necessary to analyze and explore real interactions between cryptocurrencies so that the evolution of the cryptocurrency market can be learned properly and thus investors can choose a good strategy for their investment. Moreover, the frequency resolution of our data plays an important role in the performance of correlation matrix and also community detection. Specifically, the finer the data, the more precise the community structure. Thus, we use a 30 min dataset, which is the finest available timescale in this study. The dramatic change in the community structures between bearish and bullish markets reveals a change in the investment decisions of investors. In particular, investors makes their own investment decisions based on their personal market analysis and experience during normal times. Eventually, this causes a diversification in the cryptocurrencies chosen to invest in, since not only high- but also low-ranking cryptocurrencies are added in the portfolios. On the other hand, investors tend to only trade cryptocurrencies with high market capitalization during turbulent times, while smaller cryptocurrencies are mainly used for other purposes, such as transaction fees, smart contracts tokens or simply used to run a digital platform.

## Figures and Tables

**Table 1:** A list of 34 cryptocurrencies used in this study. Abbreviations are put in parentheses.

Cryptocurrencies					
Argur (REP)	Bitcoin SV (BSV)	Ethereum Classic (ETC)	MaidSafeCoin (MAID)	Ontology (ONT)	Tron (TRX)
Bancor (BNT)	Cardano (ADA)	FunToken (FUN)	Maker (MKR)	Ox (ZRX)	Verge (XVG)
Basic Attention Token(BAT)	Decentraland (MANA)	ICON (ICX)	Monero (XMR)	QTUM	Zcash (ZEC)
Bitcoin (BTC)	Dogecoin (DOGE)	IOST	Nem (XEM)	Ripple (XRP)	Zilliqa (ZIL)
Bitcoin Cash (BCH)	EOS	Lisk (LSK)	NEO	Stellar (XLM)	
Bitcoin Gold (BTG)	Ethereum (ETH)	Litecoin (LTC)	OMG Network (OMG)	Tezos (XTZ)	

**Table 2:** Characteristics of four re-sampled datasets at four different levels of granularity.

Level of Granularity	# Data Points	# Missing Values
30 min	37,632	289 (0.8%)
6 h	3136	24 (0.8%)
12 h	1568	12 (0.8%)
24 h	784	0 (0%)

**Table 3:** Three time windows used in this work (time windows split to take into consideration the COVID-19 pandemic).

Time Window	Stage	Time Span	# Days
1	Normal time	13 February 2019–31 December 2019	322 days
2	Downturn time	1 January 2020–30 June 2020	182 days
3	Recovery time	1 July 2020–6 April 2021	280 days

**Table 4:** Cryptocurrency network connection strength through three time windows measured by Residuality Coefficient and Mean Distance. Four different granularity levels are considered, each with datasets, including original and cleaned dataset after removing noise and trend effects.

Metric	Data Type	Time Window	Granularity			
			30 min	6 h	12 h	24 h
**Residuality** **Coefficient**	Original Data	1	0.41	0.11	0.16	0.08
		2	0.28	0.111	0.06	0.05
		3	0.14	0.05	0.07	0.34
	Cleaned data	1	1.69	6.66	14.82	14.40
		2	5.98	8.90	14.41	15.34
		3	2.32	2.99	1.88	1.05
**Mean** **distance**	Original Data	1	1.08	0.82	0.80	0.76
		2	0.99	0.71	0.65	0.56
		3	0.98	0.57	0.46	0.45
	Cleaned data	1	1.29	1.38	1.42	1.42
		2	1.40	1.42	1.42	1.42
		3	1.29	1.12	1.01	1.22

**Table 5:** *v-Measure* between Louvain and Girvan–Newman methods.

	Granularity			
	30 min	6 h	12 h	24 h
**Time window 1**	0.88	1.00	1.00	1.00
**Time window 2**	1.00	1.00	1.00	1.00
**Time window 3**	0.87	0.82	0.91	1.00

**Table 6:** The growth of network structures over time measured by Betweenness Centrality and Degree Assortativity.

Metrics	Time Window 1	Time Window 2	Time Window 3
Betweenness centrality	0.15	0.05	0.16
Degree Assortativity	−0.49	−0.72	−0.51

**Table 7:** Similarity in network structures between different phases of the cryptocurrency market measured by three metrics. A higher value of v−measure indicates a greater similarity between two structures, whereas, higher values of degreecentrality and eigenvaluemethod indicate more dissimilarity between two structures.

	Time Window	1 vs. 2	1 vs. 3	2 vs. 3
Metrics				
Degree centrality		0.5	0.09	0.42
Eigenvalue method		844.45	4.59	759.16
*v*-measure		0.04	0.32	0.02

**Table 8:** Distributions of rankings in each community during different phases of the financial market: normal time, downturn time and recovery time. The rankings are sorted in ascending order. Bold values are minimum and maximum ranks in each period.

1l	Group	Cryptocurrencies	Rankings
**Normal time**	1	ADA, XLM, BAT, ZIL	10, 13, 32, 99
	2	BTG, IOST, XTZ, ZRX, ETC	12, 21, 45, 57, 83
	3	LSK, OMG, REP, FUN, MKR	26,54, 58, 70, 168
	4	NEO, MANA, BNT, XVG, XEM, QTUM	19, 31, 41, 86, 117, **184**
	5	ONT, ZEC, XMR, XRP, EOS, TRX, LTC	3, 6, 7, 11, 16, 29, 35
	6	ICX, MAID, DOGE, BTC, BSV, ETH, BCH	**1**, 2, 5, 9, 34, 84, 130
**Downturn time**	1	DOGE, ICX, BNT, MANA, ZRX, FUN, MAID, BAT, XVG, ONT	32, 33, 40, 45, 60, 81, 105, 124, 139, **196**
	2	ADA, BCH, BSV, BTC, BTG, EOS, ETH, ETC, IOST, LSK, LTC, MKR, NEO, OMG, QTUM, REP, TRX, XEM, XLM, XMR, XRP, XTZ, ZEC, ZIL	**1**, 2, 4, 5, 6, 7, 9, 11, 12, 15, 17, 18, 21, 22, 27, 30, 34, 48, 51, 53, 54, 62, 65, 91
**Recovery time**	1	BTG, MANA, BAT, ZEC	56, 62, 67, 107
	2	ONT, QTUM, EOS, BSV, MKR	24, 31, 53, 75, 88
	3	XVG, ZIL, XEM, MAID, BTC, ETH	**1**, 2, 38, 48, 109, 136
	4	ADA, DOGE, XRP, BCH, XLM, LTC	6, 7, 9, 15, 16, 20
	5	OMG, BNT, IOST, REP, ICX, LSK	68, 78, 85, 100, 101, **140**
	6	ETC, ZRX, TRX, NEO, XMR, FUN, XTZ	17, 27, 33, 35, 64, 76, 129

## Data Availability

Not applicable.
